# Enhancement of the Mechanical Properties of Hydroxyapatite/Sulphonated Poly Ether Ether Ketone Treated Layer for Orthopaedic and Dental Implant Application

**DOI:** 10.1155/2018/9607195

**Published:** 2018-08-01

**Authors:** Roohollah Sharifi, Davood Almasi, Izman Bin Sudin, Mohammed Rafiq Abdul Kadir, Ladan Jamshidy, Seyed Mojtaba Amiri, Hamid Reza Mozaffari, Maliheh Sadeghi, Fatemeh Roozbahani, Nida Iqbal

**Affiliations:** ^1^Department of Endodontics, School of Dentistry, Kermanshah University of Medical Sciences, Kermanshah, Iran; ^2^School of Dentistry, Kermanshah University of Medical Sciences, Kermanshah, Iran; ^3^Department of Manufacturing and Industrial Engineering, Faculty of Mechanical Engineering, Universiti Teknologi Malaysia, 81310 Skudai, Johor, Malaysia; ^4^Medical Devices & Technology Group (MEDITEG), Faculty of Biosciences and Medical Engineering, Universiti Teknologi Malaysia, 81310 Johor Bahru, Johor, Malaysia; ^5^Department of Prosthodontics, School of Dentistry, Kermanshah University of Medical Sciences, Kermanshah, Iran; ^6^Department of Biostatistics and Epidemiology, School of Health, Kermanshah University of Medical Sciences, Kermanshah, Iran; ^7^Department of Oral and Maxillofacial Medicine, School of Dentistry, Kermanshah University of Medical Sciences, Kermanshah, Iran; ^8^Medical Biology Research Center, Kermanshah University of Medical Sciences, Kermanshah, Iran; ^9^Bio-Medical Engineering Center, University of Engineering and Technology (UET), Lahore Kala Shah Kaku (KSK) Campus, Pakistan

## Abstract

The mechanical properties of coated layers are one of the important factors for the long-term success of orthopeadic and dental implants. In this study, the mechanical properties of the porous coated layer were examined via scratch and nanoindentation tests. The effect of compression load on the porous coated layer of sulphonated poly ether ether ketone/Hydroxyapatite was studied to determine whether it changes its mechanical properties. The water contact angle and surface roughness of the compressed coated layer were also measured. The results showed a significant increase in elastic modulus, with mean values ranging from 0.464 GPa to 1.199 GPa (p<0.05). The average scratch hardness also increased significantly from 69.9 MPa to 95.7 MPa after compression, but the surface roughness and wettability decreased significantly (p<0.05). Simple compression enhanced the mechanical properties of the sulphonated poly ether ether ketone/hydroxyapatite coated layer, and the desired mechanical properties for orthopaedic and dental implant application can be achieved.

## 1. Introduction

Success in orthopaedic and dental implant depends on several parameters that may be improved by considering both biologic and mechanical criteria [1]. The use of synthetic polymers and composites for biomaterial applications has continued to expand. Fiber-reinforced polymers offer advantages because they can be designed to match tissue properties, can be anisotropic with respect to mechanical properties, can be coated for attachment tissues, and can be fabricated at relatively low cost. Expanded future applications for orthopaedic and dental implant systems are anticipated as interest in combination synthetic and biological composites increases. The more inert polymeric biomaterials include polytetrafluoroethylene (PTFE), polyethylene terephthalate (PET), polymethylmethacrylat (PMMA), ultra-high molecular weight polyethylene (UHMW-PE), polypropylene (PP), polysulfone (PSF), and poly ether ether ketone (PEEK). These are thermal and electrical insulators, and when constituted as a high molecular weight system without plasticizers, they are relatively resistant to biodegradation [2]. The low elasticity modulus, excellent chemical stability, transparency to radio waves, and compatibility with reinforcing agents (such as carbon fiber) make poly ether ether ketone (PEEK) an ideal choice for medical applications, such as dental implants [3–5]. Despite these excellent properties, PEEK is still categorized as bioinert due to its very low reaction with the surrounding bone tissue [6]. There are several methods to improve the bioactivity of PEEK, such as selective wet-chemistry [7], grafting [8, 9], and hydroxyapatite (HA) coating [10]. In our previous studies, the bioactivity of PEEK was increased via sulphonation and deposition of HA crystalline particles on the sulphonated layer [11, 12]. The main advantage of our new method was the fact that the deposition process took place at room temperature, which caused no damage to the heat-sensitive PEEK. Our new method consists of two steps including sulphonation for activating the surface of PEEK and deposition of HA particles on the activated sulphonated PEEK (SPEEK) layer. The diffusion of sulphuric acid in the PEEK caused a porous coated layer [11]. The porous coated layer is a potential for bone interlocking; however, the modulus is expected to be relatively low for load-bearing applications such as orthopaedic and dental applications.

An adequate elastic modulus of the coating layer is important as it affects load distribution and stress shielding at the interface layer between a coated implant and the surrounding bone [13]. Also, the success of a particular implant* in vivo* depends on adequate adhesion of the coating layer [14]. Some standards must be considered for the HA coatings on medical implants, such as the thickness, porosity, roughness, pore size, and adhesion of the coating layer, to ensure the quality of their performance [15, 16]. However, the coated layer in our new type of coated layer consists of SPEEK and HA, and the existing standards are not suitable to evaluate its performance qualification.

In our previous study, we studied the feasibility of changing the mechanical property of the porous coated layer of SPEEK/HA via compression [17]. In this study, mechanical and bioactivity properties of the porous coated layer of SPEEK/HA were examined at different sulphonation times and enhanced via compression using a hydraulic press to ensure that it qualified for orthopaedic and dental implant application. Microscratch and nanoindentation tests were conducted to evaluate the mechanical properties of the coated layer. The water contact angle and surface roughness were measured. The wettability and surface roughness of the coated layer were also examined to determine the level of bioactivity of a particular material.

## 2. Materials and Methods

The PEEK substrates (Optima® Invibio) discs were ground by 400-grit silicon carbide paper. The PEEK samples were immersed in concentrated sulphuric acid (95-97%) for three different durations, 3, 5, and 10 min, in ambient temperature. The samples were then immersed with distilled water at room temperature until no traces of acid were obtained, and the SPEEK samples were left to dry at room temperature overnight. The SPEEK disc samples were then immersed in a 10% wt/v suspension of hydroxyapatite 21223 (Sigma Aldrich, USA) in water for 5 h and continuously stirred via a magnetic stirrer. After 5 h, the samples were removed, washed, and ultrasonically cleaned with deionized water for 10 min to remove any excess HA particles that were not chemically connected to the samples. The samples were then dried at room temperature overnight [11]. A compressive load of 15 MPa, equal to the ultimate compressive strength of cancellous bone [18], was applied to the surface of the coated layer via a hydraulic press for 10 minutes.

### 2.1. Nanoindentation Test

The Hysitron TI 750D Ubi nanomechanical test system with a Berkovich indenter tip was used for the nanoindentation test. Three nanoindentation tests were conducted per sample. Based on Buckle's one-tenth rule for the evaluation of the mechanical properties of a coating layer, the maximum indentation depth must be less than one-tenth of the thickness of the coating layer to prevent the effect of the substrate in the resultant force curve [19]. The maximum applied load of 200 *μ*N was chosen based on a preliminary experiment considering the one-tenth indentation rule [19]. The loading rate was chosen as 0.5 *μ*N/s, and the holding time was 300 s.

The Oliver-Pharr model was used to calculate Young's modulus of the coated layer from the indentation force curve. In nanoindentation studies of polymeric materials, the elastic properties of the indenter can be ignored due to the large differences in the elastic modulus between the tip and the sample [20]. The following Oliver-Pharr equations were based on this assumption [21]. For the Berkovich indenter, which was used in the nanomechanical test system, the projected contact area (A_c_) was obtained from the contact depth (*δ*_c_) via [22]

(1) $$\begin{array}{c} A_{c}=24.5\delta_{c}^{2} \end{array}$$

The contact depth (*δ*_c_) was obtained from (2) at the peak load (*δ*_max_), where the stiffness (S) was calculated by measuring the slope of the unloading part of the force curve at *δ*_max_.

(2) $$\begin{array}{c} \delta_{c}=\delta_{max}-\varepsilon \frac{F_{max}}{S}, \end{array}$$

where F_max_ is the load at the maximum indentation depth. The geometric constant of *ε* is 0.75 for the Berkovich indenter tip [23]. Finally, to calculate Young's modulus of the coated layer, (3) was utilized:

(3) $$\begin{array}{c} E=\frac{S\sqrt{\pi }}{2\sqrt{A_{c}}} \end{array}$$

### 2.2. Scratch Test

The progressive scratch test was carried out using microscratch test equipment from Micro Materials, Ltd. One scratch test was made per sample. The stylus speed was set at 2 *μ*m/s, with a chosen scratch length of 600 *μ*m. Normal load was not applied to the stylus for the first 60 *μ*m. The load was then increased linearly from 0 to 500 mN, with a loading rate of 2 mN/s between 60 and 560 *μ*m scratch length and remained constant in the last 40 *μ*m of the scratch length. A conical spherical Rockwell stylus with a radius of 25 *μ*m and a conical angle of 90° was used in this test. The scratch tracks were analyzed using optical microscope images during which the scratch width was measured and the coating failure point determined. The point at which the stylus reached the substrate was determined through manual observation from the optical microscope images by the changes in color from white (coating layer) to beige (substrate).

Based on the literature, there are more than 250 methods available to determine the adhesion between a coating and the coated layer [24], of which the scratch test is the most effective technique [25, 26]. The test consists of applying a continuously increasing load on the coating surface by a stylus scratching point, while the sample is displaced at a constant speed. The scratching point causes increasing elastic and plastic deformation until damage occurs in the surface region.

Based on the American Society for Testing Materials (ASTM) [27], scratch hardness (H_s_) is defined as “the normal load of the stylus over the load-bearing area”. To calculate the scratch hardness, (4) was used [27]:

(4) $$\begin{array}{c} H_{s}=\frac{4Fq}{{\pi w}^{2}}, \end{array}$$

where *F* was the normal load (in Newtons), *w* was the width of the scratch (in millimeters), and *q* was a dimensionless parameter which varied between one (for full elastic recovery of the sample) and two (for samples with no recovery). In this study, the *q* parameter was assumed to be 2 because of plastic deformation of the sample during the scratch test [27].

For the graphical determination of the scratch hardness, the plot of* F*-(*πw*^2^/4) for the sample was drawn, and the slope of the linear fit to the graph gives the scratch hardness of the material [27]. To calculate the scratch hardness of the coated layer, the data of the scratch test for the scratch distance before the critical point must be used.

Nanoscale morphology of the coated layer before and after compression and the morphology of the scratches were probed using a scanning electron microscope (SEM) (Hitachi Tabletop, TM-3000).

An atomic force microscope (AFM) (SPA-300 HV, Seiko) was used to analyze the surface roughness. The AFM was run in the force-curve mode, and a scan size of 5 *μ*m × 5 *μ*m was used to calculate the arithmetic mean of the surface roughness (Ra). Thirty lines, each with a length of 3 *μ*m, were used to calculate Ra [11].

The Sessile method was used to measure the water contact angle of the surface of the modified PEEK. Contact angle goniometer equipment (OCA 15 plus, Data Physics) was used for the measurement. The ASTM D7334-08 standard, in which deionized water was used as the liquid and the chosen drop size was 0.5 ± 0.1 *μ*l, was used in this test. For each sample, 10 points were randomly chosen from the sample's surface to measure the contact angle [11]. The data of this study were analyzed by SPSS Statistics 22 (IBM, USA) using one-way analysis of variance (ANOVA) and Tukey's test followed by post hoc least significant difference (LSD), with a significance level set at p<0.05.

## 3. Results and Discussion

### 3.1. Surface Morphology

**Figure 1:**
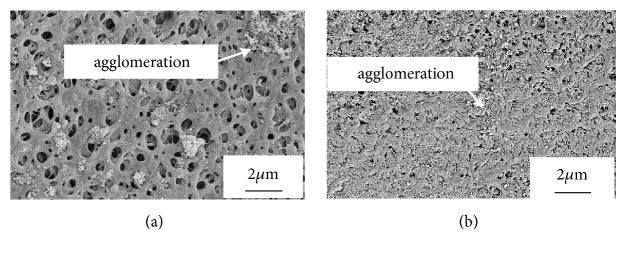
SEM image of the surface of the treated layer (a) before and (b) after applying the compression load.

Figure 1 shows the surface morphology of the coated layer with a 3 min sulphonation time before and after a 15 MPa compressive load. The load caused an increase in the density of the coated layer. Agglomeration of HA particles in the samples before compression could still be found after the load was removed, but with a reduction in size.

### 3.2. Surface Roughness

**Figure 2:**
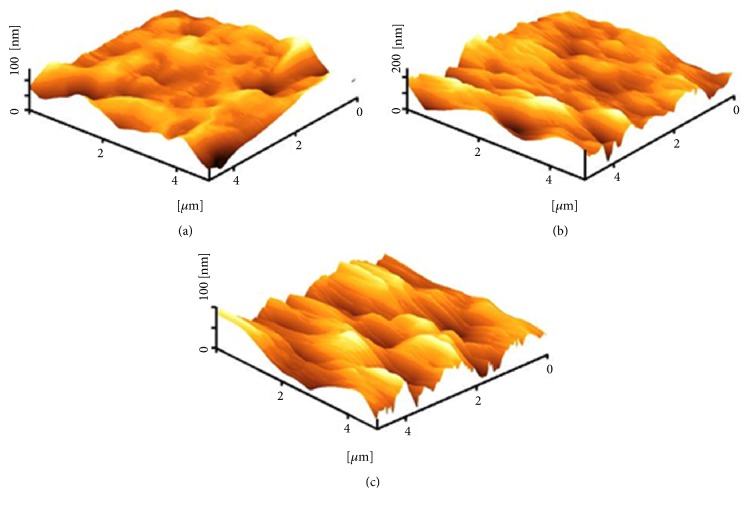
AFM 3D height images of the treated PEEK with (a) 3-, (b) 5-, and (c) 10-minute sulphonation times after applying the compression load.

Figure 2 shows the three-dimensional height image of the surface of the samples after applying the compression load via AFM. The surface morphology of all three different samples was almost the same, indicating the independence of sulphonation time on surface roughness after compression. Before compression, the surface roughness increased with increasing sulphonation time [11]. The line of the AFM tip is visible on the sample image due to the soft properties of the coated layer and the force mode which was used for this analysis. The calculated arithmetic mean of the surface roughness obtained via AFM for the compressed samples was 20.4, 22.2, and 20.9 nm for the 3, 5, and 10 min sulphonation times, respectively. These three mean values did not show significant difference (p>0.05); however, they showed significant decrease in comparison with uncompressed samples (from 34.1, 30.9, and 45.2 nm for 3, 5, and 10 min sulphonation times, respectively) [11]. This decrease in surface roughness was due to the compaction of the soft and porous coated layer under the compression load. This condition is less desirable for cell attachment and could not provide a scaffold for mechanical interlocking between mineralized bone and the implant [28].

### 3.3. Scratch Study Results and Discussions

**Figure 3:**
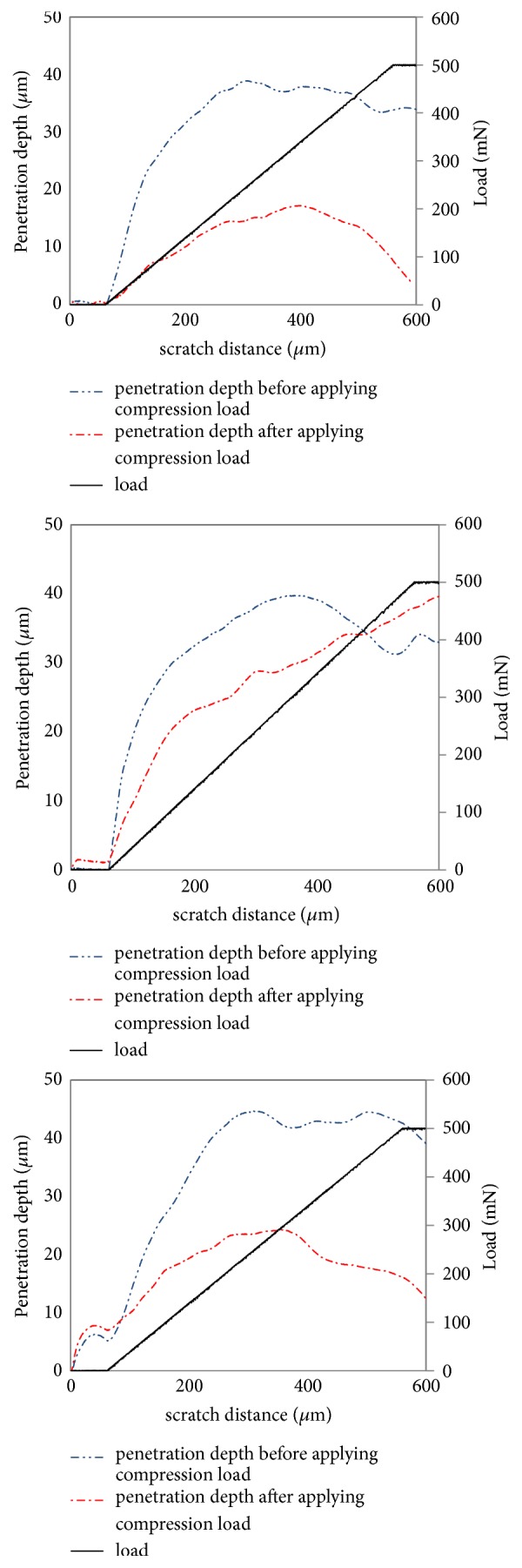
Penetration depth versus scratch distance for (a) 3-, (b) 5-, and (c) 10-minute sulphonation time without and with applying the compression load.

Figure 3 shows a graph of the penetration depth-normal load against the scratch distance of the samples. As explained above, the scratch normal load begins after 60 *μ*m from the beginning of the scratch length from 0 linearly to 500 mN at the 560 *μ*m length of the scratch and remains fixed for the last 40 *μ*m of the scratch.

Different criteria may be used to probe the failure point in the scratch test. The point of failure may be defined as the onset of microcracking, crazing, fish-scale formation, ploughing, or the point at which the coating is penetrated, revealing the underlying substrate [27]. For brittle coating layers, such as ceramics, failure occurs at two critical loads; the first is the cohesive failure at the coating layer (LC1), and the second is the adhesive failure where the load causes the coating to peel off, exposing the substrate (LC2) [29]. Cohesive failure may not occur in some soft coating layers, in which the stylus reaches straight to the substrate without any cracking, fish scaling, ploughing, etc. [27].

**Figure 4:**
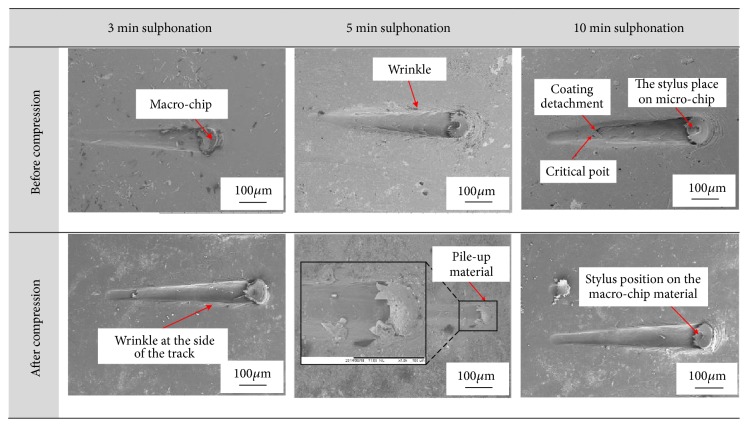
Scratch track images on the surface of the treated layer.

The penetration depth must increase with increasing normal load. However, the accumulation of the detached coating and substrate material ahead and under the stylus (macro-chip) pushes the stylus up, affecting the penetration depth. For most of the samples, the upward force caused by the macro-chip formed under the stylus (Figure 4) overcomes the effect of increasing the normal load due to the reduction of the penetration depth of the stylus at the last part of the scratch distance. Once the ultimate shear strength of the coating layer material was exceeded, plastic deformation occurred causing the materials to slip on each other, forming macro-chips. Due to the compliant property of the substrate, the macro-chips surrounding the stylus continued to remove the substrate material after full delamination of the coating. Wrinkles are also visible at the edges of the scratch track. Similar results were observed for samples after compression. The sample with 5 min sulphonation time shows a continuous increase in penetration depth as the normal load increases due to the formation of macro-chips ahead of the stylus (Figure 4). These results would be different if tested on a brittle coating layer as there would be no macro-chip material ahead or under the stylus and the only factor affecting the penetration depth would be the normal load [30, 31]. Macro-chips can only be found for the scratch testing of compliant coating layers [32].

**Figure 5:**
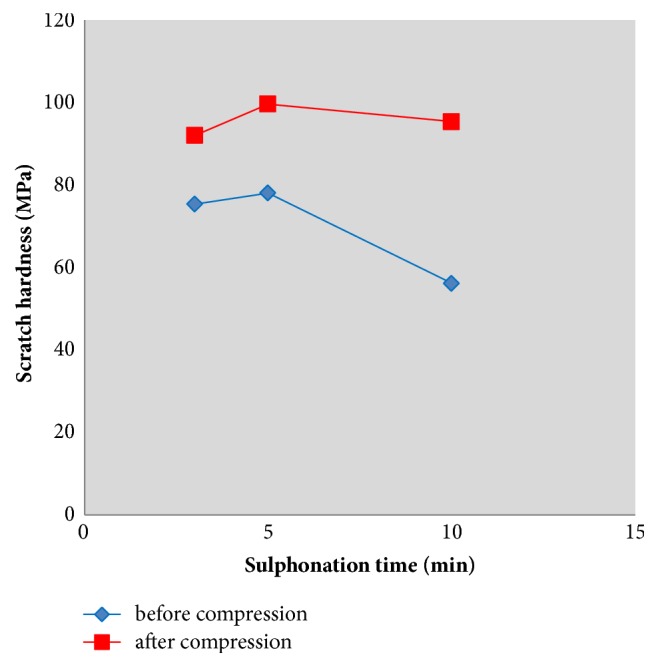
Scratch hardness of the treated layer for different sulphonation time, without and with applying compression load.

Figure 5 shows the scratch hardness measurement results on the coated layer at different sulphonation times before and after being compressed. The scratch hardness increased from 75.3, 78.1, and 56.2 MPa to 92.1, 99.6, and 95.3 MPa, respectively, after being compressed for the samples at 3, 5, and 10 min sulphonation time. The mean value of the scratch hardness of compressed samples significantly increased (36.9%) in comparison with uncompressed samples (p<0.05). The scratch hardness results indicate that compression load can be used for improving the mechanical properties of the coated layer.

**Figure 6:**
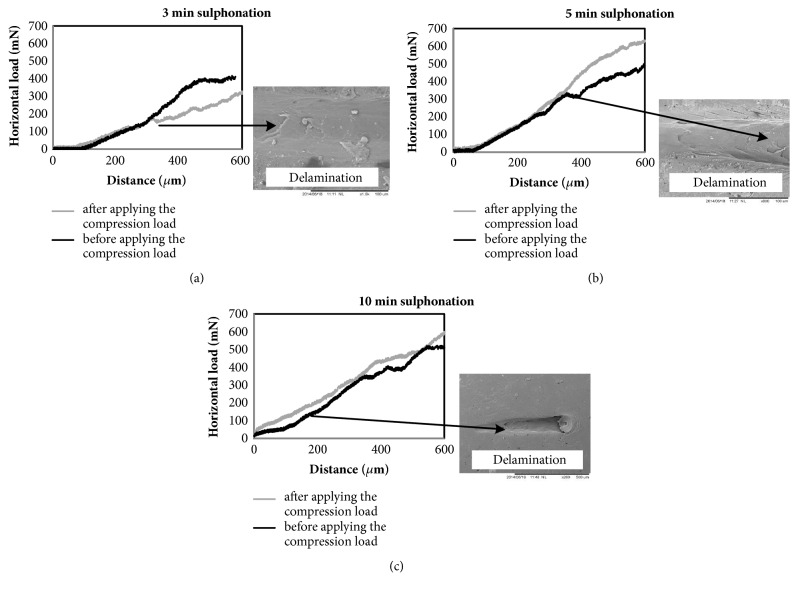
The effect of compression on the horizontal load/scratch distance with (a) 3-, (b) 5-, and (c) 10-minute sulphonation time.

Figure 6 shows the horizontal load versus scratch distance for different sulphonation times. The horizontal load increased as the applied normal load increased during the scratch test and showed an abrupt drop in load when delamination occurred on the substrate surface. In some samples, the critical point was reached with only the penetration of the stylus into the coated layer without any delamination. In these cases, no abrupt changes were observed in the horizontal load graph. The amount of the horizontal load of the first part of the horizontal load/scratch distance graph, before the critical point (before penetration of the stylus into the sample which is =200 *μ*m), is very important because it can be used to calculate the coefficient of the friction. The results showed that the horizontal load of the samples increased after compression of the coated layer. Two important factors affect the horizontal load of soft and rubbery materials, such as our coated layer: first surface roughness [33] and second elastic modulus of the coated layer [34]. As shown above, the surface roughness of the samples decreased with the applied compression which caused a reduction in the horizontal load. However, the compression changed the mechanical properties of the coated layer comprising the elastic modulus, which affected the horizontal load and resulted in the increase in the horizontal load after compression.

### 3.4. Nanoindentation Study Results and Discussions

**Figure 7:**
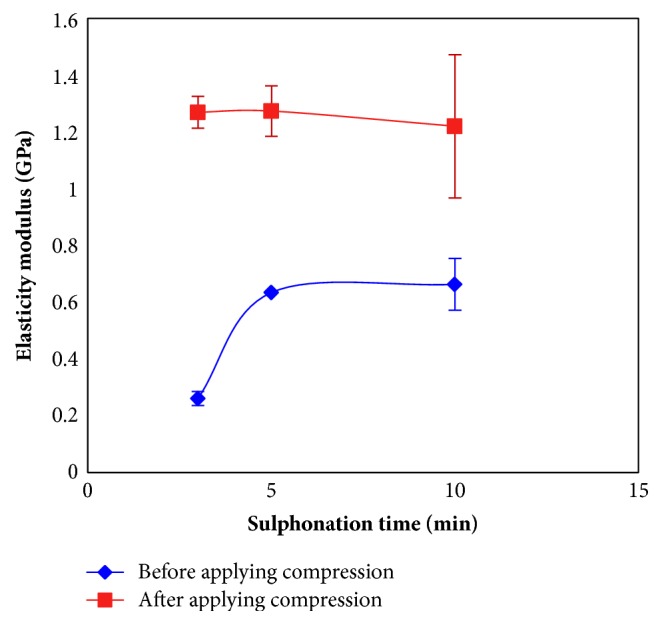
The effect of compression on the elastic modulus of a treated layer with different sulphonation times.

Figure 7 shows the modulus of elasticity of the coated layer with different sulphonation times with and without compression. The elastic modulus of the coated layer significantly increased (threefold) for 5 min compared to a 3-min sulphonation time (p<0.05). However, increasing the sulphonation time to 10 min did not increase the elastic modulus further (p>0.05). After the compression process, all samples, irrespective of the sulphonation time, produced similar magnitudes of elastic modulus. Compression caused the coated layer to become more compact. The applied compression resulted in a significant increase of the mean elasticity modulus of the coated layer from 0.464 GPa to 1.199 GPa (p<0.05). The improvement in mechanical properties can be varied to produce coated layers with different elastic moduli based on the requirements for the orthopaedic and dental implant application.

### 3.5. Water Contact Angle Analysis

**Figure 8:**
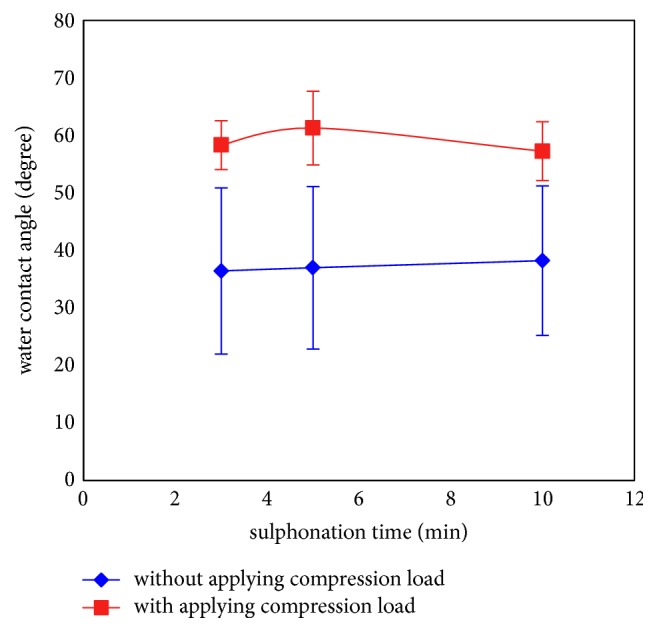
The effect of the compression load on the water contact angle of the samples with different sulphonation times [11].

Figure 8 shows the effect of sulphonation time and compression load on the water contact angle of the coated PEEK samples. The water contact angle results for the samples before applying the compression load showed a low water contact angle and the expected improvement in the wettability due to the surface treatment [11]. After applying compression load on the coated layer, the mean of the water contact angle significantly increased (59%) (p<0.05). However, this is still lower than the contact angle of bare PEEK, which is 72° [11]. It was also noticed that the water contact angle was constant with sulphonation time variations from 3 to 10 min before and after compression (p>0.05). The two main parameters of surface roughness and surface chemistry affect the water contact angle. The absorption of the water droplet traces in the compact layer (after compression), in comparison to porous surface layers before applying the compression load that permeated the water droplet, can reduce the water contact angle. However, the compression can also change the surface chemistry by increasing the water contact angle due to the soft properties of the SPEEK in comparison to HA particles. The increasing the water contact angle leads to reduced osseointegration [35], which is undesirable.

## 4. Conclusion

The nanoindentation and scratch hardness study revealed the sulphonation time did not have a uniform trend in mechanical properties of coated layer. The elastic modulus of coated layer increased via increasing the sulphonation time from 3 to 5 minutes; however, increasing the sulphonation time to 10 minutes did not increase the elastic modulus further. The scratch hardness of the coated layer increased via increasing the sulphonation time from 3 to 5 minutes; however, increasing the sulphonation time to 10 minutes decreased the scratch hardness. The applied compression resulted in a significant increase of mean elasticity modulus of coated layer from 0.464 GPa to 1.199 GPa, and enhanced the mean scratch hardness of the samples from 69.9 MPa to 95.7 MPa, as the porosity was reduced through compaction. After compression, the surface roughness decreased due to the compaction of the porous coated layer. The mean elastic modulus of the coated layer for different sulphonation times increased from 0.464 GPa to 1.199 GPa. The water contact angle for 3, 5, and 10 min sulphonation times after compression increased from 37.2° to 58.9°, which is lower than the value for the bare PEEK of 72°. The mechanical properties of PEEK with chemical deposition of HA on its surface can be enhanced through simple compression and reach the requirements for orthopaedic and dental implant application. However, the improvement comes at the expense of achieving lower wettability.

## Data Availability

The data used to support the findings of this study are available from the corresponding author upon request.
